# Sympathetic Overactivity and Parasympathetic Impairment in Type 2 Diabetes: An Analysis of Cardiovascular Autonomic Functions

**DOI:** 10.7759/cureus.59561

**Published:** 2024-05-03

**Authors:** Amrutha Kanagala, J. M. Harsoda

**Affiliations:** 1 Department of Physiology, Smt. B. K. Shah Medical Institute and Research Centre, Sumandeep Vidyapeeth, Deemed to be University, Vadodara, IND

**Keywords:** parasympathetic regulation, sympathetic activity, blood pressure, heart rate, cardiovascular autonomic dysfunction, t2dm

## Abstract

Background

Cardiovascular autonomic dysregulation is a known complication of Type 2 diabetes mellitus (T2DM), characterized by dysregulation in heart rate (HR) and blood pressure (BP). These disruptions in cardiovascular autonomic control can significantly influence the morbidity and mortality associated with the disease.

Objectives

This study aims to investigate how T2DM affects cardiovascular autonomic functions by comparing responses in HR, BP, and specific autonomic function tests between a control group without diabetes and a study group with diabetes. The research questions focus on assessing HR variability, baroreflex sensitivity, and other autonomic parameters to determine the extent of cardiovascular autonomic dysregulation in diabetic patients.

Methods

This cross-sectional study involved 200 adults, divided equally between a control group (n = 100) and a T2DM study group (n = 100). The exclusion criteria included cardiovascular diseases and renal impairment. Data collection involved assessing baseline characteristics such as age and BMI. Cardiovascular measures, including HR, systolic blood pressure (SBP), and diastolic blood pressure (DBP), were recorded after a five-minute rest. Autonomic function tests assessed sympathetic and parasympathetic responses, including the cold pressor test and the isometric hand grip exercise test. The statistical analysis was conducted using IBM SPSS Statistics for Windows, Version 25 (Released 2017; IBM Corp., Armonk, New York, United States), focusing on independent t-tests to compare between groups, considering p-values <0.05 as significant. Potential confounding variables like age and BMI were accounted for in the analysis to ensure robust findings

Results

The study group showed a higher average BMI (28.95 ± 5.60) compared to the control group (26.50 ± 5.70) and an increased resting HR (74.20 ± 8.60 bpm vs. 69.30 ± 9.10 bpm). The SBP was slightly higher in the study group (115.00 ± 19.00 mmHg vs. 114.50 ± 8.90 mmHg), while the DBP was lower (71.50 ± 10.70 mmHg vs. 72.80 ± 6.70 mmHg). The autonomic function tests showed a smaller increase in SBP (106.80 ± 11.00 mmHg) and a larger increase in DBP (75.90 ± 8.30 mmHg) upon standing in the study group compared to controls. The cold pressor test indicated increased sympathetic activity in the study group, with significant rises in SBP (133.70 ± 10.30 mmHg) and DBP (83.40 ± 9.00 mmHg) compared to the control group (SBP: 114.31 ± 11.87 mmHg, DBP: 71.85 ± 8.67 mmHg). These findings demonstrate marked differences in cardiovascular autonomic responses between the groups.

Conclusions

This study demonstrates that T2DM significantly impacts cardiovascular autonomic functions, with diabetic patients showing altered HR and BP indicative of increased sympathetic and decreased parasympathetic activity. These autonomic dysfunctions may heighten cardiovascular risk in diabetic individuals. Our findings highlight the importance of monitoring and managing cardiovascular autonomic functions in diabetic patients to reduce their risk of cardiovascular complications. Further research should investigate the underlying mechanisms and the effectiveness of interventions to improve autonomic function in this population.

## Introduction

Type 2 diabetes mellitus (T2DM) is a chronic metabolic disorder characterized by insulin resistance and insufficient insulin production, leading to elevated blood glucose levels. The global prevalence of T2DM has reached alarming proportions, affecting over 422 million individuals worldwide, and is projected to increase to 642 million by 2040 [1]. This escalating prevalence is particularly concerning given the significant impact of T2DM on global morbidity and mortality rates, especially through cardiovascular complications. Cardiovascular diseases remain the primary cause of death in T2DM patients, accounting for approximately 50% of all deaths in this group [2].

Cardiovascular autonomic dysfunction is a notable complication of T2DM, though it often goes unrecognized in clinical settings. This condition involves the impairment of the autonomic nervous system, which is integral to regulating cardiovascular functions such as heart rate (HR) and blood pressure (BP). The autonomic nervous system is divided into the sympathetic and parasympathetic nervous systems. The sympathetic nervous system responds to stress by accelerating HR and raising BP, whereas the parasympathetic nervous system helps the body rest and digest, slowing down the HR [3]. Dysregulation in these systems can lead to various cardiovascular issues, including decreased heart rate variability (HRV) and orthostatic hypotension. While orthostatic hypotension, causing a sharp drop in BP upon standing and potentially leading to fainting and falls, is a crucial indicator of cardiovascular health, HRV represents the variation in the time interval between heartbeats [4,5].

The potentially serious consequences of improper management of cardiovascular autonomic dysfunction in T2DM serve as a reminder of how important it is to understand this condition. Undetected or poorly managed autonomic dysfunction can precede and predict severe cardiovascular events, such as myocardial infarction, sudden cardiac death, and stroke [6]. Finding and treating these autonomic irregularities early on can greatly improve the outcomes for T2DM patients by lowering the number and severity of these events [7,8].

Despite the critical nature of this complication, there remains a substantial knowledge gap in the comparative analysis of autonomic functions between individuals with T2DM and healthy controls. Furthermore, the effectiveness of interventions specifically targeting autonomic dysfunction in T2DM patients has not been thoroughly explored [7,8]. This study seeks to address these gaps by conducting detailed comparisons of autonomic function tests between diabetic and non-diabetic individuals. Such research is essential for deepening our understanding of T2DM’s impact on cardiovascular health and refining intervention strategies to enhance patient care and prevent severe cardiovascular complications.

## Materials and methods

Study setting and period

The Department of Physiology at the Mamata Medical College in Khammam, India conducted this cross-sectional study after receiving approval from the Institutional Ethics Committee (IEC) under IRB/IEC No. 94. The study was conducted from January 2022 to December 2023, a period chosen to minimize seasonal effects on the collected data and to capitalize on increased participant availability following the normalization of healthcare services post-pandemic.

Participants

The study included 200 adult participants, strategically divided into two equal groups to enable a robust comparative analysis. The control group comprised 100 healthy individuals without T2DM, while the study group consisted of 100 patients diagnosed with T2DM, as per the American Diabetes Association's (ADA's) criteria. This specific number of participants was determined through statistical power calculations to ensure the study had enough power to detect significant differences in cardiovascular autonomic functions between the two groups, with a 95% confidence level and 80% power, aiming to minimize Type I and Type II errors and enhance the reliability of the results. To control potential confounding variables that could affect the study outcomes, exclusion criteria were applied rigorously; individuals with cardiovascular diseases, renal impairment, or other conditions known to influence autonomic functions were not included. All participants provided written informed consent, ensuring they were fully informed about the study's procedures and potential risks, upholding ethical standards, and protecting participant rights throughout the research process.

Data collection

Baseline characteristics such as age and BMI were meticulously gathered following standardized protocols to ensure consistency and accuracy. BMI was measured using a calibrated digital scale for weight and a stadiometer for height, with participants wearing minimal clothing and no shoes. Resting cardiovascular measures, including HR, SBP, and DBP, were assessed after a five-minute rest period to stabilize cardiovascular parameters. BP was measured using a calibrated aneroid sphygmomanometer, and both auscultatory and palpatory methods were employed to ensure precise readings. The auscultatory method, involving the use of a stethoscope to listen for Korotkoff sounds, was primarily used to obtain the most accurate systolic and diastolic measurements. The palpatory method was also implemented initially to estimate SBP before using the stethoscope. This helped in determining the appropriate cuff inflation level, thus enhancing the accuracy of the subsequent auscultatory measurements.

A suite of autonomic function tests was selected to evaluate both sympathetic and parasympathetic functions comprehensively. The immediate standing test was used to assess postural changes in BP and HR, which helps in evaluating the integrity of the reflex arc involved in cardiovascular responses to sudden positional changes, indicating parasympathetic function. The cold pressor test was employed to assess the sympathetic stress response, involving the immersion of one hand in ice-cold water to observe changes in BP and HR. This test is particularly useful for assessing stress-induced sympathetic activation. Additionally, the isometric hand grip exercise test was conducted to further evaluate sympathetic function. This test involves maintaining a handgrip at 30% of maximum voluntary contraction for a few minutes to measure the cardiovascular response to sustained muscular contraction. These tests were administered under strict protocols to ensure consistency and reliability. For the immediate standing test, participants were initially rested in a supine position for five minutes before standing, with measurements taken before and immediately after the posture change. In the cold pressor test, baseline cardiovascular measurements were recorded before immersing the hand in cold water for up to 90 seconds, with further readings during and after immersion to track changes. The isometric hand grip exercise test required participants to grip a dynamometer at a predetermined level of their maximum capacity, with cardiovascular monitoring throughout the exertion and recovery period. These tests were chosen because they effectively reveal the functional status of both branches of the autonomic nervous system, providing insights into the balance between sympathetic and parasympathetic activities in individuals with and without T2DM.

Instruments and measurements

The instruments used in our study, including the aneroid sphygmomanometer and standard electrocardiogram (ECG) for measuring BP and HR respectively, are widely recognized for their clinical accuracy and reliability. The aneroid sphygmomanometer is calibrated regularly against a mercury sphygmomanometer to ensure its precision, adhering to the standards set by the American Heart Association (AHA). The ECG equipment used for monitoring HR is routinely tested and calibrated to maintain adherence to manufacturer specifications and international clinical guidelines, ensuring its validity and reliability in capturing precise cardiac rhythms.

We made specific adaptations to the standard protocols to enhance the accuracy of our measurements and to tailor the procedures to the needs of our study population. For BP measurements, we incorporated a preliminary palpatory method to determine the appropriate level of cuff inflation before employing the auscultatory method, which is not always standard practice but was deemed necessary to prevent underestimation or overestimation of BP, especially in diabetic patients who may have arterial stiffness. Additionally, during the ECG recording for HR measurement, we extended the resting period prior to recording from the typical three to five minutes to five to seven minutes to ensure a more stable baseline, considering the potential variability in autonomic function among participants with T2DM.

These adaptations were carefully documented and implemented to maintain the integrity and accuracy of our data collection process, ensuring that the modifications enhanced our ability to capture precise and clinically relevant measurements. These details underline our commitment to methodological rigor and transparency in our research practices.

Statistical analysis

These data were evaluated using IBM SPSS Statistics for Windows, Version 25 (Released 2017; IBM Corp., Armonk, New York, United States). Descriptive statistics, including means and standard deviations, were calculated for all baseline characteristics and outcome measures. Comparative analysis between the control and study groups was performed using independent t-tests for continuous variables to identify significant differences in cardiovascular and autonomic function parameters. The level of significance was set at p < 0.05. Where applicable, chi-square tests were used for categorical data to assess differences in proportions. To address potential confounding factors such as age, BMI, and the duration of diabetes, we conducted multivariate regression analyses. These adjustments helped in isolating the effects of T2DM from other influencing factors. Additionally, sensitivity analyses were performed to assess the robustness of our findings against variations in data handling and analysis methods. Subgroup analyses were also carried out to explore differences within cohorts defined by specific demographic and clinical characteristics, further refining our understanding of the data. These analyses ensure that our conclusions are not only statistically significant but also relevant and reliable across different patient profiles and under various analytical conditions.

Ethical considerations

The approval for the study's protocol was granted by the IEC at the Mamata Medical College. Participants received information regarding the objectives and methods of the study, and written informed consent was secured before collecting any data. 

We adhered strictly to ethical guidelines to ensure the confidentiality and privacy of all participants throughout the study. Personal information was securely collected and stored in a database accessible only to authorized research staff. Each participant was assigned a unique identifier code, and all study-related documents and data files used these codes instead of personal identifiers. Additionally, all electronic data were encrypted and stored on secure servers to ensure that no individual participant could be identified.

To minimize potential risks and discomfort, all study procedures were designed to be as non-invasive as possible. Participants were thoroughly informed about the nature of the tests involved, such as the immediate standing test, cold pressor test, and isometric hand grip exercise test, and what to expect during each procedure. Trained medical personnel monitored participants throughout these tests to ensure their safety and comfort. If any undue stress or discomfort was observed, the test was immediately stopped, and appropriate medical attention was provided. All participants provided informed consent before participating, having been fully informed of any potential risks and their right to withdraw from the study at any time without consequences.

These measures were implemented not only to protect the rights and well-being of our participants but also to maintain the integrity of the study and ensure the validity of the collected data.

## Results

The current study aimed to assess the impact of T2DM on cardiovascular autonomic functions by comparing cardiovascular measures and autonomic responses between a control group (n = 100) and a diabetic group (n = 100). We conducted a detailed analysis of baseline characteristics, resting cardiovascular measures, and sympathetic and parasympathetic function test responses.

Baseline characteristics

There was no significant difference in age between the diabetic group (46.89 ± 7.60 years) and the control group (48.75 ± 9.65 years), indicating minimal age-related influence on our findings. However, BMI was significantly higher in the diabetic group (28.95 ± 5.60) compared to the control group (26.50 ± 5.70; p < 0.05). This suggests a correlation between a higher BMI and the presence of T2DM. These baseline characteristics are summarized in Table 1.

**Table 1:** Baseline Characteristics BMI: body mass index

Parameters	Control Group Mean ± SD	Study Group Mean ± SD
Age (Years)	48.75 ± 9.65	46.89 ± 7.60
BMI	26.50 ± 5.70	28.95 ± 5.60

Resting cardiovascular measures

Our analysis revealed significant differences in cardiovascular measures at rest. The diabetic group exhibited a higher mean HR (74.20 ± 8.60 bpm) compared to the control group (69.30 ± 9.10 bpm; p < 0.05). There was also a slight but not statistically significant increase in SBP in the diabetic group (115.00 ± 19.00 mmHg) versus the control group (114.50 ± 8.90 mmHg). DBP, however, was significantly lower in the diabetic group (71.50 ± 10.70 mmHg) compared to the control group (72.80 ± 6.70 mmHg; p < 0.05). These results indicate that T2DM may be associated with alterations in resting cardiovascular states, potentially contributing to cardiovascular risks. These findings suggest that T2DM may be associated with alterations in resting cardiovascular states, as detailed in Table 2.

**Table 2:** Resting Cardiovascular Measures HR: heart rate; SBP: systolic blood pressure; DBP: diastolic blood pressure

Parameters	Control Group Mean ± SD	Study Group Mean ± SD
HR (bpm)	69.30 ± 9.10	74.20 ± 8.60
SBP (mmHg)	114.50 ± 8.90	115.00 ± 19.00
DBP (mmHg)	72.80 ± 6.70	71.50 ± 10.70

The higher resting HR observed in the diabetic group can be indicative of decreased HRV, which is a recognized marker of poor cardiovascular health. Reduced HRV is associated with an increased risk of cardiac events, as it reflects reduced heart adaptability to stress. The consistently higher HR in the diabetic group suggests chronic sympathetic overactivation, which can lead to cardiac overload and potentially increase the risk of heart failure.

Sympathetic function test parameters

The sympathetic function tests highlighted significant differences. Upon immediate standing, the diabetic group showed a smaller increase in SBP (106.80 ± 11.00 mmHg) than the control group (112.00 ± 12.20 mmHg), indicating diminished sympathetic responsiveness (p < 0.05). Conversely, DBP increased significantly more in the diabetic group (75.90 ± 8.30 mmHg) compared to the control group (67.90 ± 8.20 mmHg; p < 0.05). During the cold pressor test, the diabetic group exhibited significantly higher increases in both SBP (133.70 ± 10.30 mmHg) and DBP (83.40 ± 9.00 mmHg) than the control group, suggesting heightened sympathetic activity (p < 0.01). These results are encapsulated in Table 3.

**Table 3:** Sympathetic Function Test Parameters SBP: systolic blood pressure; DBP: diastolic blood pressure

Parameters	Control Group Mean ± SD	Study Group Mean ± SD
SBP (mmHg)	112.00 ± 12.20	106.80 ± 11.00
DBP (mmHg)	67.90 ± 8.20	75.90 ± 8.30
Cold Pressor Test - SBP (mmHg)	114.31 ± 11.87	133.70 ± 10.30
Cold Pressor Test - DBP (mmHg)	71.85 ± 8.67	83.40 ± 9.00

The differences in BP responses, especially the lower DBP in resting conditions and the exaggerated response during the cold pressor test in the diabetic group, highlight significant vascular dysfunction. In diabetes, chronic hyperglycemia can lead to vascular stiffness and altered endothelial function, which impairs the body's ability to regulate BP effectively. This dysfunction can increase the risk of developing hypertension and subsequent cardiovascular complications such as stroke and coronary artery disease.

Parasympathetic function test parameters

The evaluation of parasympathetic function revealed a significant increase in HR upon immediate standing in the diabetic group (100.20 ± 10.40 bpm) compared to the control group (88.00 ± 10.60 bpm; p < 0.01). This indicates impaired parasympathetic regulation in the diabetic group, reflecting potential challenges in cardiovascular stress adaptation (Table 4). 

**Table 4:** Parasympathetic Function Test HR: heart rate

Parameters	Control Group Mean ± SD	Study Group Mean ± SD
Immediate Standing - HR (bpm)	88.00 ± 10.60	100.20 ± 10.40

The significant changes observed during the sympathetic and parasympathetic function tests underline the imbalance in autonomic nervous system regulation. Diabetic autonomic neuropathy, a common complication of diabetes, can manifest as such an imbalance, leading to unpredictable cardiovascular responses to physical stress or postural changes. This can increase the risk of sudden cardiac death in diabetic patients.

## Discussion

The goal of this study was to find out how T2DM affects cardiovascular autonomic functions by looking at the differences in HR, BP, and responses to autonomic function tests between healthy controls and people with T2DM. The study results show that T2DM has a significant effect on the heart's autonomic functions. HR, BP responses, and autonomic functions were very different between diabetic patients and healthy controls. The higher resting HR observed in the diabetic group could indicate reduced HRV, which is a marker of diminished cardiovascular health and an increased risk of cardiac events due to chronic sympathetic overactivation. Also, the fact that a diabetic's BP responses are different, especially their lower diastolic pressure at rest and higher response during stress tests, suggests that their blood vessels are not working properly, and they cannot control their BP as well as they should. This could raise their risk of high BP and the complications that come with it. The big differences between the sympathetic and parasympathetic function tests show an autonomic imbalance that is common in people with diabetic autonomic neuropathy. This can cause unpredictable heart responses and raise the risk of sudden cardiac death.

The higher resting HR and different BP responses seen in the diabetic group align with earlier research that suggests T2DM is linked to problems with the autonomic nervous system [9,10]. For example, the elevated resting HR could reflect a compensatory mechanism to maintain cardiac output in the face of diabetic cardiac autonomic neuropathy [11-13]. In the same way, the different ways that BP responded to tests of autonomic function in our study show that baroreflex sensitivity is low, which is a sign of cardiovascular autonomic dysfunction in people with diabetes [14,15].

The fact that the study group's BP went up a lot during the cold pressor test and the isometric hand grip exercise test shows that people with T2DM have more sympathetic activity [16,17]. This is consistent with the notion that chronic hyperglycemia leads to increased sympathetic tone, contributing to the risk of hypertension and cardiovascular complications [17].

These results back up Osailan's research from 2021, which talks about how important autonomic imbalance is in the development of cardiovascular diseases in diabetic groups [18]. Additionally, our research contributes to the growing evidence indicating that autonomic dysfunction in diabetes extends beyond parasympathetic withdrawal to include increased sympathetic activity [19].

The demonstration of autonomic dysfunction in individuals with T2DM underscores the importance of early screening and intervention. Autonomic testing could be integrated into routine diabetes care to identify individuals at high risk of cardiovascular complications. Moreover, interventions aimed at improving glycemic control and lifestyle modifications could be pivotal in mitigating autonomic dysfunction and its associated risks [20].

Several potential confounding factors need to be considered, including the higher BMI observed in the diabetic group. This factor is associated with altered autonomic functions and could overlap the effects of diabetes. Although age differences were not statistically significant, the duration of diabetes, which often correlates with age, could affect autonomic functions, suggesting that longer disease duration might exacerbate autonomic dysfunction. Additionally, undiagnosed comorbidities such as cardiovascular diseases or renal impairments could also skew the results, necessitating comprehensive screening to isolate the effects of diabetes.

These observations highlight the clinical importance of routine autonomic function assessments in diabetic patients, not only for diagnosis but also for the early identification of those at high risk of cardiovascular complications. Such assessments can guide targeted interventions, including lifestyle changes and strict glycemic control, to improve autonomic balance and reduce cardiovascular risks. Future research should focus on longitudinal studies to track autonomic function over time and evaluate the impact of specific interventions, thus providing clearer insights into the causal relationships and the efficacy of treatment strategies in improving cardiovascular health in diabetic patients. This approach emphasizes the need for a comprehensive cardiovascular autonomic evaluation in diabetes management protocols to mitigate the heightened risk of cardiovascular diseases in this population.

Limitations

The limitations of this study are crucial to understand for interpreting the findings accurately and considering their application to broader populations. One significant limitation is the cross-sectional nature of the study, which restricts our ability to infer causality from the observed associations between T2DM and cardiovascular autonomic dysfunction. Additionally, the study was conducted at a single center, which may limit the generalizability of the findings to other populations with different demographic or healthcare characteristics.

Furthermore, while we controlled for some confounding factors such as age and BMI, other potential sources of bias could include the duration of diabetes, the level of glycemic control, and the presence of other comorbid conditions, which were not thoroughly examined. These factors could significantly influence the autonomic function and cardiovascular health of the participants, thus potentially biasing the study results. For example, a longer duration of diabetes is generally associated with more severe complications, which could exacerbate autonomic dysfunction. Similarly, varying levels of glycemic control among participants could influence the severity of cardiovascular autonomic dysfunction observed.

Future directions

Building on the insights gained from this study on the impact of T2DM on cardiovascular autonomic functions, future research should prioritize longitudinal studies to establish causal relationships and track the progression of autonomic dysfunction over time. Expanding participant diversity by including multiple centers and diverse populations will enhance the generalizability of findings. It is also crucial to conduct a comprehensive assessment of potential confounding variables such as the duration of diabetes, glycemic control levels, and the presence of other metabolic syndromes. Implementing advanced technologies for more precise autonomic function tests and integrating data analytics could provide deeper insights into the autonomic nervous system's responses in diabetic patients. Interventional studies designed to evaluate the effectiveness of specific treatments aimed at improving autonomic function will be valuable, as would research focusing on patient-centered outcomes such as quality of life and the frequency of diabetic complications. Additionally, exploring the underlying biological mechanisms through basic science and clinical studies could elucidate how diabetes affects cardiovascular autonomic functions. These future directions will refine our understanding and enhance the clinical management of diabetes, aiming to improve overall outcomes for patients with T2DM.

## Conclusions

This research highlights the profound impact of T2DM on cardiovascular autonomic functions, revealing significant variances in HR and BP responses between individuals with T2DM and those without the disease. These findings indicate that diabetic individuals exhibit increased sympathetic activity and diminished parasympathetic regulation, predisposing them to an elevated risk of cardiovascular issues that could exacerbate morbidity and mortality. These insights underscore the critical importance of early detection and tailored therapeutic strategies. Integrating systematic autonomic function testing into routine diabetic patient assessments could facilitate earlier intervention. Implementing such measures may enhance autonomic regulation and diminish the incidence of cardiovascular complications, thus supporting the inclusion of comprehensive autonomic function evaluations in diabetes management protocols.
